# A new method of finding groups of coexpressed genes and conditions of coexpression

**DOI:** 10.1186/s12859-016-1356-3

**Published:** 2016-11-25

**Authors:** Rajat Anand, Srikanth Ravichandran, Samrat Chatterjee

**Affiliations:** 1 Drug Discovery Research Centre, Translational Health Science and Technology Institute, NCR Biotech science cluster, 3rd milestone, Faridabad-Gurgaon expressway, Faridabad, 121001 India; 2 Immunology group, International Centre for Genetic Engineering and Biotechnology, Aruna Asaf Ali Marg, New Delhi, 110067 India

## Abstract

**Background:**

To study a biological phenomenon such as finding mechanism of disease, common methodology is to generate the microarray data in different relevant conditions and find groups of genes co-expressed across conditions from such data. These groups might enable us to find biological processes involved in a disease condition. However, more detailed understanding can be made when information of a biological process associated with a particular condition is obtained from the data. Many algorithms are available which finds groups of co-expressed genes and associated conditions of co-expression that can help finding processes associated with particular condition. However, these algorithms depend on different input parameters for generating groups. For real datasets, it is difficult to use these algorithms due to unknown values of these parameters.

**Results:**

We present here an algorithm, clustered groups, which finds groups of co-expressed genes and conditions of co-expression with minimal input from user. We used random datasets to derive a cutoff on the basis of which we filtered the resultant groups and showed that this can improve the relevance of obtained groups. We showed that the proposed algorithm performs better than other known algorithms on both real and synthetic datasets. We have also shown its application on a temporal microarray dataset by extracting biclusters and biological information hidden in those biclusters.

**Conclusions:**

Clustered groups is an algorithm which finds groups of co-expressed genes and conditions of co-expression using only a single parameter. We have shown that it works better than other existing algorithms. It can be used to find these groups in different data types such as microarray, proteomics, metabolomics etc.

**Electronic supplementary material:**

The online version of this article (doi:10.1186/s12859-016-1356-3) contains supplementary material, which is available to authorized users.

## Background

To capture the behavior of an organism under different experimental conditions, we need a method that simultaneously study and compare the gene/protein expression level measured for different conditions (e.g. time points, tissue types) [1]. High-throughput techniques like microarray [2, 3] and recently RNA-seq techniques [4] are used to measure mRNA levels of all genes in the genome of a studied organism across a range of conditions of an experiment. In such high throughput data, instead of looking at the expression levels of each gene separately, it is more informative to look at the groups of genes coexpressed across conditions, since they may represent a biological process [5]. Moreover, in a microarray data where conditions are time points, linking perturbed biological processes temporally can help us relating initial perturbed biological processes with the processes perturbed at later time points. A common method to extract such clusters from a high-throughput data is ‘clustering’ [5, 6]. Another extension of such method is ‘biclustering’ which is useful to capture the genes that are correlated only in a subset of samples [7].

Many algorithms have been introduced since the year 2000, which extract groups of co-expressed genes and associated conditions of co-expression from a microarray data. Few of them are CC algorithm [7], ISA algorithm [8], BiMax algorithm [9], SAMBA algorithm [10] and QUBIC algorithm [11]. These algorithms require different input parameters for generating biclusters from high throughput microarray data. This is the first limitation of these algorithms. On a real dataset, it is difficult to know apriori about the values of these parameters to find the biclusters and hence, wrong input parameter may lead to wrong result. Hence an algorithm with very less parameters, preferably no parameter, is expected to be more useful on real datasets. The second limitation of the existing algorithms is that none of them have been tested on the data (real or synthetic) where the biclusters are overlapping in presence of noise. These algorithms were tested on synthetic dataset with implanted biclusters and real datasets with known biclusters and compared their performance to recover implanted or known biclusters [9, 12, 13]. The algorithms were evaluated on datasets with increase in noise levels. But, these synthetic dataset did not have any overlapping biclusters, which is very common in real biological data.

To overcome these two limitations, we introduce a new algorithm which uses only one parameter: depending on whether we want overlapping biclusters in the result or not. Accordingly, we set the parameter equal to 1 for overlapping bicluster and 0 for non-overlapping. In our algorithm, we first discretize each gene and then group them based on their similar discretized profiles. Finally, we select clusters (or groups) with high correlation coefficient and large size. These high correlation coefficient clusters along with the discretization information gives the biclusters with both genes and conditions. Our method is similar to ‘Correlation maximization biclustering methods (CMB)’ which seeks for subsets of genes and samples where the expression values of the genes (samples) correlate highly among the samples (genes) [13]. Other CMB algorithm such as CC algorithm [7] also uses similar method to extract biclusters by imposing the condition that the mean square residue is below some threshold value δ.

In the present paper, we first introduce the algorithm and then show its performance on synthetic and real datasets. We then compare our algorithm with other existing algorithms from literature. Finally, we show the application of our algorithm on a real biological dataset obtained from a mouse liver tissue.

## Methods

### Algorithm to find groups of co-expressed genes and conditions of co-expression

Given a microarray mRNA data matrix with *N* number of genes across *C* number of conditions, we need to find groups of co-expressed genes and the conditions of co-expression. We first started with each gene separately and determined the conditions where it is expressed. We used the idea similar to one proposed by [14] where the whole set of expression values of a gene across conditions is used to determine whether the gene is perturbed or not. However, in [14], the set of conditions where the gene is perturbed was not determined. To take care of this limitation, we modified the above idea by using the first difference formula (detailed below) for each gene separately resulting in discretization of genes. We then grouped genes based on their discretization pattern resulting in clusters. The clusters with high correlation coefficient were further combined resulting in a set of clusters. We then described each obtained clusters by two parameters: cluster size (number of genes) and correlation coefficient (correlation of genes within the cluster). Next, we need to derive a cutoff on these two parameters to filter biclusters with high correlation coefficient and high size. For this, we considered a matrix of genes with expression randomly generated from a normal distribution with mean zero and a given variance. We calculated the values of these two parameters (cluster size and correlation coefficient) using the biclusters obtained from this random matrix and derived a cutoff (detailed below) on these two parameters, which is used to filter biclusters.

Below, we are presenting the steps of the algorithm one by one in detail. For better understanding of the algorithm we are providing a small dataset example depicting application of each step of our algorithm. To enable readers to replicate our results, a small dataset example with its output bicluster results has been provided as Additional file 1: File S1 and output of each step of our algorithm on dataset is depicted in Additional file 2: Figure: S1. For reader’s benefit, the steps of the algorithm are presented in the following format as different functions as done in [11, 15].

#### Step1 (DISCRETIZE function)

**Fig. 1:**
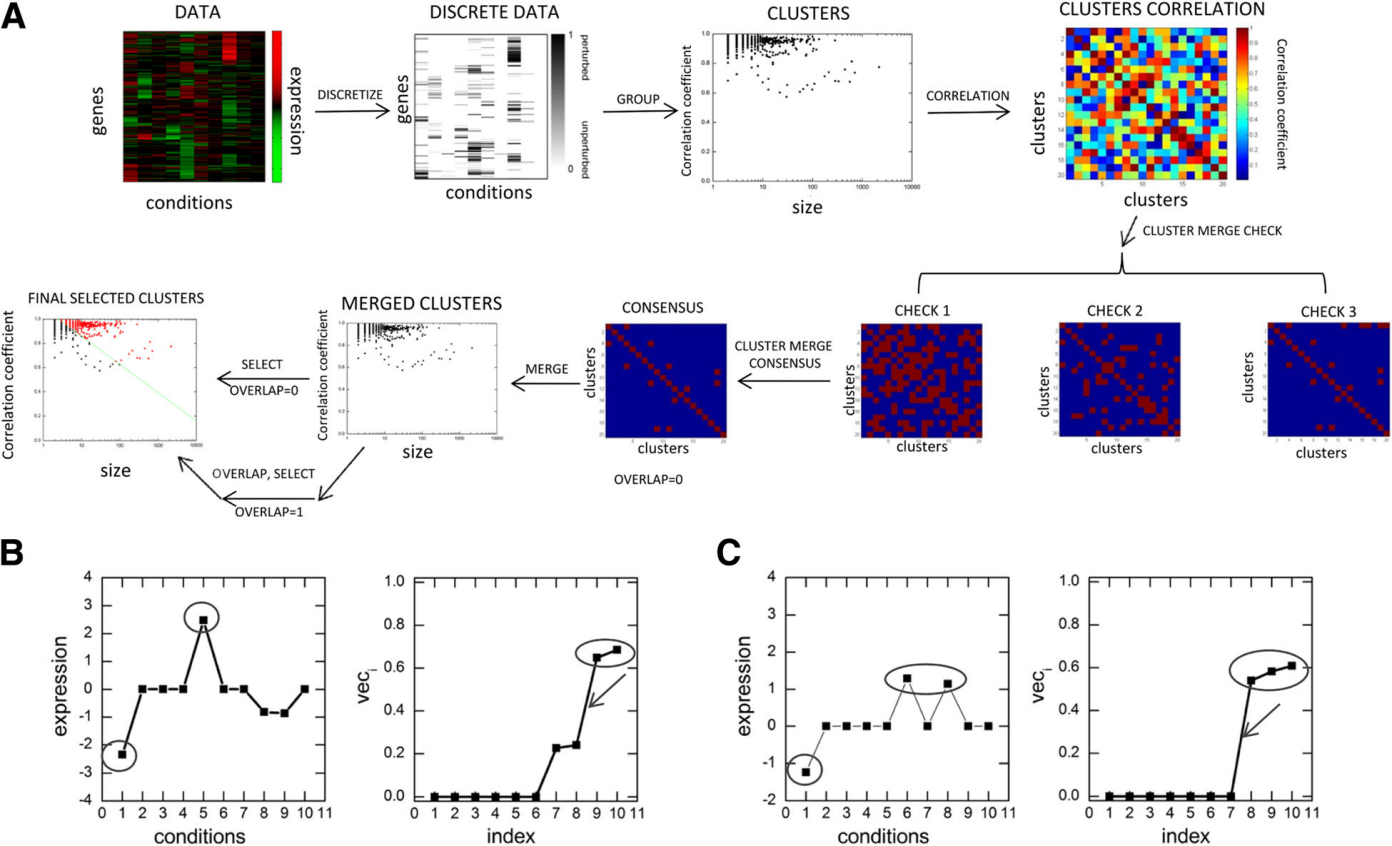
Outline of the algorithm. **a** Flowchart of the algorithm is shown with different steps explained in text. **b**, **c** The DISCRETIZE step of the algorithm is shown on two representative genes. Here, the expression level of two genes at different conditions taken from an experimental data is plotted in left panels in B and C. In right panels, the sorted absolute normalized values of expression data for the two respective genes are plotted. Arrows correspond to the point above which the expression values are characterized as expressed by the algorithm and the corresponding expression values are shown by circled values here and in actual expression data in left panels in B and C. This point is captured by taking the consecutive differences of sorted absolute normalized expression profile and identifying the index where this difference is maximum

Given a microarray mRNA data for *N* number of genes across *C* number of conditions, we first normalized the data by dividing each gene by the square root of the sum of the squares of their expression across the conditions. To find groups of co-expressed genes and the conditions where this co-expression occurs, we first need to find the condition(s) where each gene is expressed. For this, we used expression profile (i.e. expression of a gene across all the conditions) of each gene separately, e.g. gene ‘*a*’ and identified the condition(s) where gene ‘*a*’ shows high expression value relative to other condition(s). These condition(s) are obtained by taking the consecutive differences of sorted absolute normalized expression profile of gene ‘*a*’. This helps us to identify the index with maximum difference. The condition(s) corresponding to this identified index and all other indices above this index gives us the condition(s) where the gene is expressed. This step is illustrated in Fig. 1b and c for two genes with selected conditions circled in left panels and corresponding indices encircled in right panels. Some more example genes are provided in Additional file 3: Figure S2. This is the DISCRETIZE function of the algorithm outlined in Fig. 1a, which discretizes each profile (rows in the DATA matrix) one by one to transform the DATA matrix to a DISCRETE DATA matrix.

#### Step 2 (GROUP function)

Once we have the discretized data, in the next step, we group them into different clusters using the GROUP function. After discretizing each gene based on its expression values, we added the sign of expression i.e. positive or negative depending on its up or down regulation. Then, we grouped genes with same signed discretized profiles into a cluster. Each cluster is shown in a 2-D plot (Fig. 1a) by a point where its coordinates are size (i.e., the number of genes present in the cluster) and correlation coefficient (i.e., the average correlation coefficient between expression profiles of each pair of genes in the cluster).

#### Step 3 (CORRELATION function)

This step of the algorithm generates cluster correlation matrix which is used to find cluster pairs sufficiently similar to each other and hence could be merged. We termed this step as CORRELATION function. We used this step on the clusters (obtained using the GROUP function) to get the cluster correlation matrix (details in Correlation coefficient within and between clusters). The cluster coefficient matrix contains values of correlation coefficients within and between the clusters. Here, we made negative correlation values zero to ensure that the corresponding pairs don’t come as correlated pairs. Each row of this matrix contains set of correlation values of each cluster with all other clusters which we call correlation profile of the cluster. In the next step, we used the CLUSTER MERGE CHECK function on this correlation matrix to find cluster pairs with close correlation coefficients.

#### Step 4 (CLUSTER MERGE CHECK function)

In this step we have three independent checkpoints which decides if two clusters can be merged for further analysis or not.

#### Checkpoint1 (CHECK1)

In this step, we first discretized the cluster correlation matrix using our DISCRETIZE function (Step 1). This gives, for each query cluster, a set of clusters obtained after discretizing its correlation profile. We then selected those cluster pairs which are present in the discretized correlation profile of each other. Say, for an example, we picked clusters *x* and *y*, if cluster *y* is present in the discretized correlation profile of cluster *x*, and vice-versa. Thus, CHECK 1 filters cluster pairs, as shown in the red dots in the matrix in Fig. 1a (CHECK1).

#### Checkpoint2 (CHECK2)

In this step, we multiplied the correlation profile for each pair of clusters (input cluster pair) and discretize the product of correlation profiles of the cluster pairs. Finally, we checked if the cluster(s) obtained are same as the input cluster pair or not. If yes, then those two clusters are filtered through CHECK2 function, as shown in red dots in the matrix in Fig. 1a (CHECK2).

#### Checkpoint3 (CHECK3)

Here, for each pair of clusters, we checked if the average correlation coefficient between them is greater than the minimum correlation of each cluster with itself or not (for details see Section 3.2). If so, then that pair of clusters were filtered through CHECK3, as shown in red dots in the matrix in Fig. 1a (CHECK3).

The cluster pairs satisfying all these three checks are termed as CONSENSUS clusters pairs and are merged using the next step of the algorithm called MERGE function.

#### Step 5 (MERGE function)

In this step, we group the clusters pairs found above by taking the union of the genes and union of the conditions of the paired clusters. This gives us the merged clusters. This step of the algorithm that groups the cluster pairs is termed as MERGE function.

#### Step 6 (OVERLAP function)

This step provides the user with a choice to get an overlapping bicluster. This step is termed as OVERLAP function. Here, the user chooses a predefined parameter (termed as overlap parameter), which gives them the choice to go for overlapping bicluster (see Section 3.3 for details). If the user selects overlap parameter equal to 1, then the biclusters are allowed to overlap and we go to the next step for final selection of cluster. If overlap is selected as zero, then we directly go to the next step for final selection of cluster.

#### Step 7 (SELECT function)

**Fig. 2:**
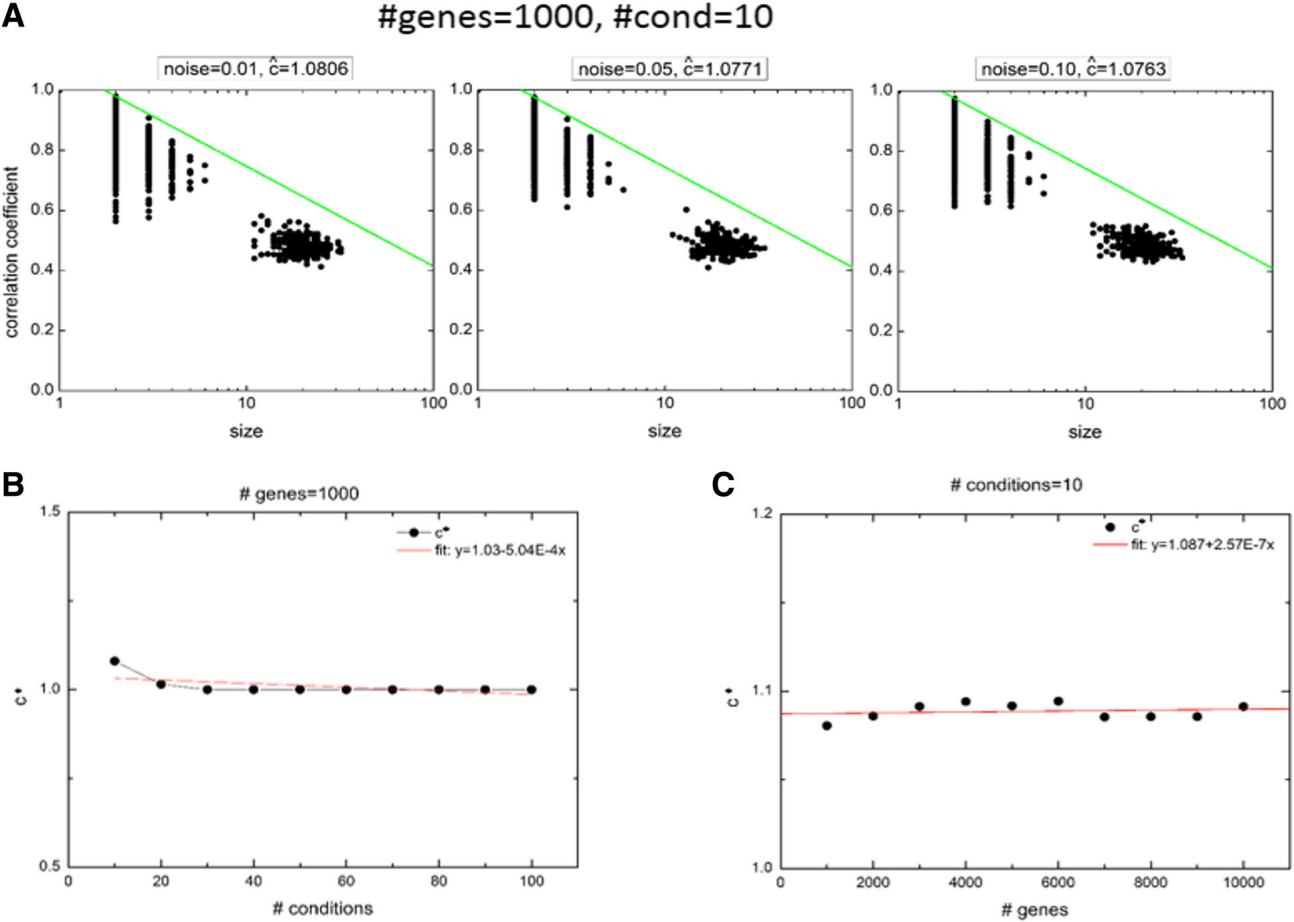
Derivation of score cutoff of clusters. **a** For an Insilco data with fixed noise level, the size and correlation coefficient of output clusters for each of the 10 runs from the algorithm are overlaid on each other and are shown in first subplot. Three subplots correspond to three noise levels in input data matrices. And the cutoff line is shown in green. The clusters sizes and correlation coefficient plot doesn’t change much for different noise levels. **b** The value of c* as a function of number of conditions in input matrix is shown showing no significant change with number of conditions as shown by the fit with *R2* = 0.3 suggesting a good fit as straight line with slope zero and intercept as mean. **c** The value of c* as a function of number of genes in input matrix is shown showing no significant change of c* with number of genes as shown by the fit with *R2* = 0.0290 suggesting a good fit as straight line with slope zero and intercept as mean

In a real dataset, due to it complex patterns, normally we get clusters with different sizes and different correlation coefficients. The clusters with large size and high correlation coefficient will contain a large number of genes showing similar pattern and could be relevant in terms of some biological process. Whereas, the clusters with small size and/or low correlation coefficient can be considered as the random clusters with very less or no functional relevance. So, in this step of the algorithm which we termed as SELECT function, we separate the relevant clusters from the random ones by using a cutoff. To derive the cutoff, we first generated the clusters by applying our algorithm on randomly generated genes and checked their cluster size and correlation coefficient values. We then checked whether, the genes with expression values generated randomly from a normal distribution could form a cluster with large size and high correlation coefficient. For this, we built random data matrices, each of 1000 × 10 dimension (made with expression values of 1000 genes under 10 conditions) where the gene expression values were generated from a normal distribution with mean zero and standard deviation equal to different noise levels. The same procedure was followed as given in Section 3.4, except that in this case no pattern was considered for the genes. We applied our algorithm on these 1000 × 10 data matrices with three noise levels (0.01, 0.05, 0.10) and plotted the size and correlation coefficient of the resultant clusters in three subplots, see Fig. 2a. In each subplot, results of the algorithm output for 10 different realizations of the input matrix were overlaid. We observed that with increase in the size of clusters, there was a decrease in the correlation coefficient and this pattern was same for all the input matrices generated with different noise levels, Fig. 2a. Since a major aim of the present study is to use minimum parameter(s), in order to filter relevant clusters from random clusters, so we used a straight line (quantified with one parameter, as derived below) as shown by green colored line in Fig. 2a. We also used dataset with different number of genes and conditions to check how much this parameter is varying with size of input dataset. When we varied condition values upto 10 fold (keeping genes values fixed), we observed no change in the cluster size (Additional file 4: Figure S3A), but increasing gene values (again upto 10 fold) and keeping condition values fixed, we notice increase in the cluster size (Additional file 4: Figure S3B). This implied that the parameter depends only on the number of genes in input dataset. Thus, to get the parameters associated with the straight line to be independent of size of input dataset, we normalized the cluster sizes with a factor log (*N*), where *N* is the number of genes in the input dataset. The resulting straight line with normalizing factor is shown below:

1 $$ y+ \log (x)/ \log (N)=c $$

where *y* is the correlation coefficient, *x* is the size of the bicluster, *N* is the number of genes in the input matrix and *c* is the parameter that can be obtain using random matrix data.

Since a real dataset contains unknown noise level, so we need to derive a threshold value for *c* (say c*), which is independent of the noise level. For a given random data matrix, applying the algorithm, we get say *K* numbers of biclusters, each having fixed *x* and *y*. We then calculated *c* using Eq.(1) for each *x* and *y*, and defined $\overline{c}= \max \left({c}_i\right)\;i=1:K.$

To obtain the value of parameter c* for a given *N X C* dimensional real dataset matrix, where *N* is the number of genes and *C* is the number of conditions, we first generated random data matrix of same dimension for a fixed noise level (as given in left subplot of Fig. 2a) and obtained $\overline{c}$. We obtained 10 such $\overline{c}$’s after generating 10 such data matrices and took $c= \max \left({\overline{c}}_i\right)i=1:10$. We repeated the above exercise for two more noise levels (as given in middle and right subplot of Fig. 2a) and obtained required *c* ^∗^ = max(*ĉ*_*i*_)*i* = 1 : 3.

Finally, we need to check that the value of c* is independent of the number of genes and number of conditions of the input data matrix. We measured the value of c* using the input data matrices of different sizes as mentioned above (resulting biclusters are shown in plots in Additional file 4: Figure S3). We fitted a straight line to the data of c* versus *C* (Fig. 2b) and obtained a fit with equation c* = 1.03-5.04E-4C with *R2* = 0.3. This low *R2* value implies that the best fit straight line is the line with zero slope and an intercept equal to mean of the data. Similarly, fitting a straight line to the data of c* versus *N* in Fig. 2c gave a linear fit with *R2* = 0.0290. This low *R2* value again implies that the best fit line is the line with zero slope and an intercept equal to mean of data. Thus, obtained c* is independent of the dimension of input matrix and hence can be used to filter the resultant biclusters.

### Correlation coefficient within and between clusters

Correlation coefficient of a cluster with itself was calculated by taking pairwise dot product of normalized expression profiles of all its constituent genes. Then, mean of the resultant set was taken. Correlation coefficient of a cluster with another cluster was defined in similar way: taking dot product of normalized expression profiles of all pairwise genes i.e. one gene of the pair was taken from cluster 1 and the second gene of the pair from cluster 2 and finally, mean of the resultant was taken.

### Overlap between biclusters

Till now, the algorithm gives different biclusters with no gene overlapping but samples can be overlapping. If the user allows the overlapping i.e. taking overlap parameter equal to 1, we followed the following procedure resulting in biclusters with gene overlap too. For each query bicluster, we found bicluster(s) (resulting biclusters) that contained samples of query bicluster. If the number of such resulting biclusters were greater than zero, then we took the union (intersection) of genes (conditions) of query and resulting biclusters to create an overlapping bicluster. If we didn’t find any biclusters, we searched for biclusters (resulting biclusters) whose samples were subset of samples of this query bicluster. If the union of samples of resulting biclusters is smaller than that of query bicluster, we include query bicluster in the list of overlapping biclusters. This procedure was repeated for each query bicluster.

### Generation of Insilco data

A matrix of zeros was created with 100 rows and 100 columns denoting 100 genes and 100 samples respectively with 1st 10 genes upregulated at 1st 10 samples i.e. expression value of these genes at these samples is 1. Similarly, the next 10 genes are up at next 10 samples. This was repeated and we get a pattern of 10 × 10 sub matrix block at the diagonal of the original matrix. These 10 X 10 sub matrix blocks represent ideal biclusters to be used to calculate recovery and relevance scores. Then normal distributed random numbers with mean 0 and standard deviation as per the noise levels given in text were added to the matrix to generate the final matrix. Same procedure was followed for the case where biclusters were overlapping; the expression value at overlap region remaining 1. For data with zeros noise and non-zero noise with overlapping clusters, we used a 100 X 100 matrix. For data with non-zero noise and non-overlapping clusters, we used 100X 50 matrix with ten 10 X 5 blocks at diagonal of matrix.

### Assembling real data

The real data was collected from human gene expression data series in NCBI GEO database (http://www.ncbi.nlm.nih.gov/geo/) with GEO accession GSE2361. It contains expression of all genes of human across 36 normal tissues. TiGER database [16] was used to collect the tissue specificity information of each gene. Only genes unique to a particular tissue were used resulting in gene tissue relationship and expression profiles of the genes belonging to Brain, Colon, Heart, Kidney, Liver, Placenta, and Testis were used for analysis. The cancer dataset was taken from GDS3716.

### Processing of microarray data

The microarray data were obtained from an experiment where one group of mice were fed with high fat high sucrose diet (HFHSD) (treated group) and another group with normal diet (control group) for certain days before taking tissue samples from both the groups of mice. Both groups of mice were fed respective diets in the following days: Day1, Day 6, Day 10, Day 14, Week 0, Week 3, Week 6, Week 9, Week 12, Week 15 and Week 18. This experiment was repeated for three times. Then, microarray experiment was performed on tissue samples and after suitable normalization of the signal intensities of each probe using Agilent Genespring GX software, three values of log fold change for the control sample and the treated sample were obtained for each probe and at each time for each tissue. Further details of the experiment are given in [17].

This data for liver tissue was downloaded from the NCBI repository under GEO accession number GSE63175. The data also contains information about data pertaining to mice fed with high fat high sucrose diet plus an ayurvedic formulation which is out of scope from our present study. The data of the ayurvedic formulation corresponds to the columns with columns header “P2_HFx_y “(x: 5,20,75 and y contains time point and sample number information) and were removed. The column headers have information of the time point of the experiment in days as well as weeks. Weeks were recorded in the experiment as the number of weeks after Day 14. Thus, 14 days were added while converting weeks to days. This implies Day 14 and Week 0 would correspond to same time and thus the information of the Day 14 and Week 0 was combined in the final matrix. So, the final time points in the matrix are Day 1, Day6, Day 10, Day 14, Day 35, Day 56, Day 77, Day 98, Day 119, and Day 140.

For each probe, the means of the log fold change for treated samples were calculated and a p-value signifying difference between three control values and three treated values by *t*-test was generated. The data contains 40628 probes corresponding to 29411 gene symbols. Gene symbol information for each probe was taken from column with column header “Gene symbol”. There can be multiple probes corresponding to a gene. We used the following steps to obtain a single value for each gene:

- Step 1. First, we filtered the data to have only those genes whose absolute values are changed for at least 2 fold in all three treated samples at a time point i.e. whose values (log) are lying outside the interval (−1 and 1) and considered them significantly perturbed genes. In case two probes corresponding to the same gene satisfy this condition, the probe with minimum p-value was chosen. We repeated this process for data at different time points and combined (union) all filtered genes to form a matrix of filtered genes and time points. The matrix elements are fold change values of all filtered genes inserted at respective time points. If a gene is not significantly perturbed at some time point, then the matrix element of that gene at that time point will be empty. For these genes, we used the following steps to insert values at these time points.
- Step 2. For the selected genes with empty matrix element at some time point, we check its probe’s fold change value at all three samples. If all these values are outside the interval (−1 and 1), we select those probes and go to step 3. If no probe of the selected gene satisfies the above criteria, we then select the probes which would have values at all three samples within the interval (−1 and 1) and go to Step 3.
- Step 3. The selected probe’s average over three samples were taken if in all three cases the value is greater/less than 0. If multiple probes of a gene satisfied this condition, probe with minimum p-value was chosen. If no probe out of selected probes satisfies this condition, the probe’s average value over two (out of three) samples with value greater/less than 0 was taken. For multiple probes satisfying this condition, probe with minimum p-value was taken. For the probe chosen, if the average value lied between −0.8 to 0.8, then for simplicity we inserted a number 0.001 in the matrix, else the average value was inserted.

The resulting matrix contained log fold change values at eleven time points. We combined Day 14 and Week 0 information in the following way. If a gene is significantly perturbed (in same direction) for both time points, then we took the average value. If they are perturbed in opposite directions, we assigned a small number (0.001) to that gene. If the gene’s value is perturbed at only one time point, we used that value in the matrix. If it is unperturbed at both the time points, we assigned any one of the non-perturbed value in the matrix. In the resulting matrix of 10 time points, if a gene is not perturbed even at a single time point, it is removed.

The resulting matrix contained log fold change values at ten time points for 19303 genes. The matrix was clustered using default clustergram function of MATLAB which uses algorithm of Eisen et al. [5] resulting in heatmap shown in Fig. 5A.

## Results

### Benchmarking the algorithm

Here, we have benchmarked our algorithm based on two scores: recovery and relevance scores. The scores were compared with best performing algorithms (as shown in [12]) like ISA [8], Bimax [9], Qubic [11], SAMBA [10] and CC algorithm [7] algorithm [7]. Though there exist other algorithms in the literature like Jaisri et al. [18] and Tesson et al. [15], but these are not considered in the present study as Jaisi et al. [18] have applied normal clustering algorithm and Tesson et al. [15] finds differential co-expressed modules between two conditions only. For this benchmarking exercise, we used two synthetic datasets and two real datasets. In the Synthetic Dataset1, instead of using the SELECT function to filter biclusters, we used all clusters. SELECT function is more useful in complex datasets to filter off clusters of small size and low correlation coefficient, for example, in clusters containing noisy genes. Application of this is shown in section (4.1.2) with Synthetic Dataset2. For the two real datasets, we used the first as the tissue dataset obtained from GSE2361 and second as the cancer dataset from GDS3716. BIMAX algorithm was run using BICCLUST R PACKAGE [19]. QUBIC algorithm was run in R by importing the package [20]. ISA AND CC were run in BICAT PACKAGE [21] while SAMBA was run using EXPANDER [22].

#### Synthetic Dataset 1

**Fig. 3:**
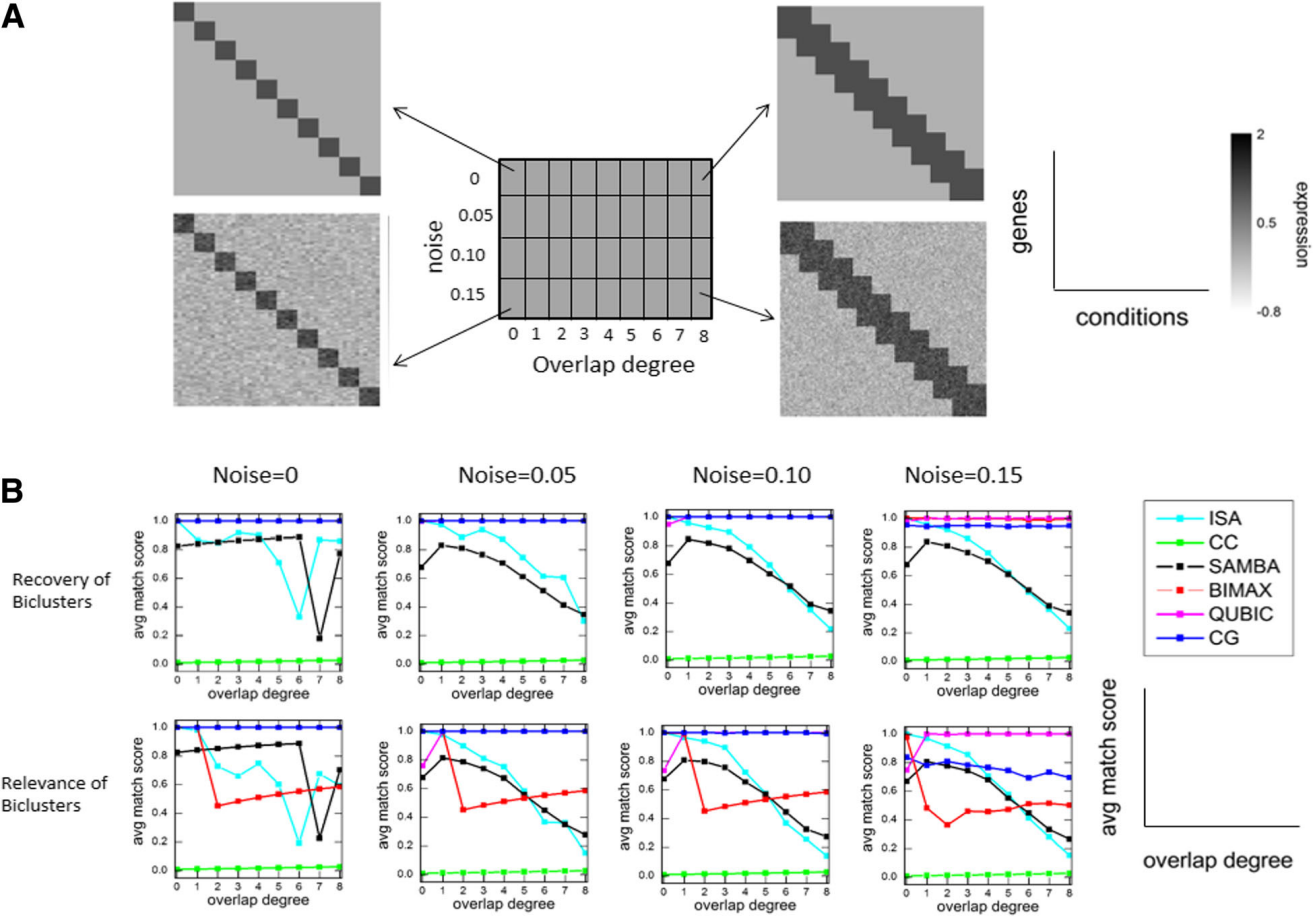
Application of algorithm to Insilco data and comparison with other algorithms. **a** A schematic showing different Insilco datasets on which algorithms are applied. Different datasets had different levels of noise and overlap degree. **b** Comparison with other algorithms when applied on Insilco datasets as shown above. Both rows contain different graphs when algorithms are applied on different datasets with increasing noise levels (column wise graphs). In first row, in each graph recovery of biclusters score is plotted as a function of overlap degree of biclusters in datasets on which algorithm is applied. Lower row contains graphs where relevance of biclusters score is plotted. CG algorithm performs better in each scenario. In the recovery graphs, qubic and bimax algorithm overlap completely with CG algorithm and can’t be seen here. Similarly, in the relevance graphs, qubic algorithm overlap completely with CG algorithm (upto noise = 0.10) and hence can’t be seen here

We used the same strategy as mentioned in Eren et al. [12], where biclusters were implanted in a background noisy matrix and the ability of different algorithms were evaluated to recover implanted biclusters. Here also we implanted overlapped biclusters, with different degree of overlap, in a background noisy matrix (see Materials and Methods: Generation of Insilco data). Synthetic datasets are shown in Fig. 3a where actual/implanted biclusters are clearly visible. We applied different algorithms (ISA, Bimax, Qubic, SAMBA and CC) on synthetic datasets and generated the output biclusters. Comparing the output biclusters with the actual implanted biclusters, we obtained two scores quantifying ability of the algorithm to recover known biclusters and also the relevance of obtained biclusters.

We used a score *S*(*M*_1_, *M*_2_) comparing two bicluster sets M1 and M2 as given in Eren et al. [12]:

$$ S\left({M}_1,{M}_2\right)=\frac{1}{\left|{M}_1\right|}{\displaystyle \sum_{b_1\in {M}_1}\underset{b_2\in {M}_2}{ \max \kern0.5em s\left({b}_1,{b}_2\right)}} $$

where |*M*_1_| is the number of biclusters in bicluster set *M*_1_.

Here, *s*(*b*_1_, *b*_2_) is chosen to be the Jaccard coefficient applied to matrix elements defined by each biclusters as given in Eren et al. [12]:

$$ s\left({b}_1,{b}_2\right)=\frac{\left|{b}_1\cap {b}_2\right|}{\left|{b}_1\cup {b}_2\right|} $$,

where |*b*_1_ ∩ *b*_2_| is the number of elements in intersection of two biclusters i.e. number of intersecting genes X Number of intersecting conditions common between two biclusters *b*_1_, *b*_2_ and |*b*_1_ ∪ *b*_2_| is the number of elements in their union. *S* takes values between 0 and 1, where 0 means two biclusters are disjoint and 1 means biclusters are identical. Any score between 0 and 1 is the fraction of total elements shared by both biclusters.

Let *E* be the set of actual biclusters and *F* be the set of output biclusters from the algorithm. Recovery score is calculated as *S*(*E*, *F*); its maximum value being 1 implies *E* ⊆ *F* i.e. algorithm has captured all the ideal biclusters. Relevance score is calculated as *S*(*E*, *F*); its maximum value being 1 implies *F* ⊆ *E* i.e. all found biclusters were true biclusters.

For a dataset of fixed noise levels and fixed overlap degree, we generated 10 data matrices, and on each data matrix we applied different algorithms to capture biclusters. As a control, we first obtained actual biclusters from the data matrices before adding noise (see Materials and Methods: Generation of Insilco data). We then compared the resulting biclusters with the actual biclusters and calculated scores using the above formulas. Thus, for each fixed noise level and overlap degree, we obtained 10 recovery and relevance scores for each algorithm. We then took their mean. These mean values obtained for matrices of different noise levels and overlap degrees were plotted in Fig. 3b. From Fig. 3b, it is clear that our algorithm (Clustered Groups (CG)) performs better than other algorithms in all cases except in case of QUBIC algorithm where it performs equivalent to QUBIC algorithm. For noise = 0.15, the relevance of CG algorithm is slightly low. In this case, the extra biclusters found by the algorithm can be removed by using the SELECT function of the algorithm and the relevance can be improved as shown in next section.

#### Synthetic Dataset 2

To show the importance of the SELECT function in improving the relevance of the obtained biclusters, we here considered a more complex dataset. We used an input dataset similar to Fig. 3A with different overlap degrees and a fixed noise level = 0.15 (maximum noise level considered in our study). We added same number of noisy genes as in the original matrix and the resulting matrices are shown in Additional file 5: Figure S4A. For each data matrix of fixed overlap degree, we applied our CG algorithm with and without the above SELECT function and compared with QUBIC algorithm. We then obtained 10 recovery and relevance scores corresponding to 10 runs and calculated their mean values. We obtained such mean scores for data matrices with different overlap degrees and plotted them in Additional file 5: Figure S4B. The results clearly suggest that the recovery scores do not change with the SELECT function but there is an increase in relevance scores after applying SELECT function and is now much better than QUBIC algorithm. We want to mention here that we obtained recovery scores is close to 1, even in the presence of noisy genes. This suggests that we have successfully filtered pure biclusters. Next, we tried to understand why this is happening. For this, we applied our algorithm on a matrix containing 1000 genes in 10 conditions with expression values generated from a normal distribution with mean zero and standard deviation equal to the noise level = 0.15. We checked the number of genes present in each biclusters and plotted them against the number of conditions associated with the corresponding bicluster. We observed that with the increase in the number of conditions in a bicluster, there is a decrease in the size of that bicluster (Additional file 6: Figure S5). This clearly suggests that there is a less chance of obtaining a bicluster having more than 2 genes and 6 conditions. Thus, the chance of presence of noisy genes in a well-defined bicluster is very less, which is also observed in the high recovery scores we obtained in Additional file 5: Figure S4B.

#### Real Dataset 1

**Fig. 4:**
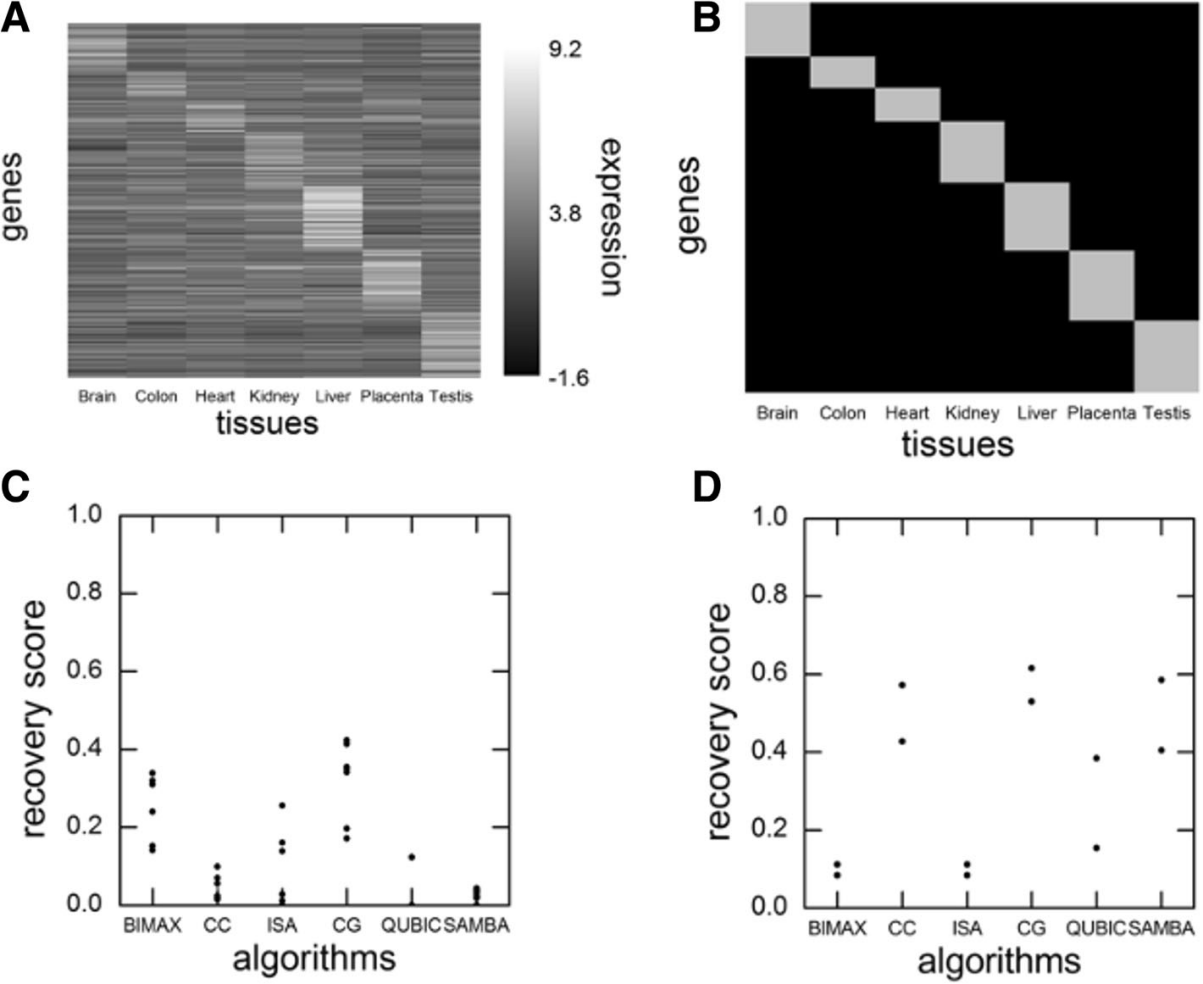
Application of algorithm to real data and comparison with other algorithms. **a** A heatmap showing expression levels of genes across tissues. **b** Actual biclusters according to classification from TiGER database are shown as gray rectangles along diagonal of matrix. **c** For each algorithm, 7 dots are shown which correspond to 7 actual biclusters. Dots represent the maximum similarity of each actual bicluster when compared with biclusters found by algorithms i.e. individual recovery scores. Average of these 7 scores gives net recovery score. **d** Plot same as in (**c**) except the input dataset used is the cancer dataset as explained in text and recovery scores to recover cancer samples are calculated

For real datasets, we followed the methodology mentioned in Oghabian et al. [13]. Here, we considered predefined biclusters and then evaluated the ability of different algorithms to recover these predefined biclusters. We used the TiGER database [16] which contains tissue specific gene list. Therefore, here, our actual biclusters are defined by TiGER database. We also downloaded the expression levels of all human genes across all tissues as profiled by Microarray (see Materials and Methods: Assembling Real Data). From this matrix of expression values, we selected only those genes whose tissue specificity is mentioned in TiGER database. Therefore, we have a matrix of expression levels of these genes specific to different tissues. The resulting matrix of genes versus tissues is shown as heatmap in Fig. 4a where we can visually identify the biclusters (biclusters are presented as diagonal blocks in Fig. 4b). Here also we followed the same procedure as done for Synthetic Dataset. Figure 4c shows the individual recovery scores for each algorithm and here also we can observe that CG algorithm performs the best.

#### Real Dataset 2

As a final benchmarking of our algorithm, we used a real dataset from the breast tumor dataset GDS3716 and compared our algorithm’s performance with performance of other algorithms. The dataset and the comparison strategy is similar to one used in Oghabian et al. [13]. The dataset contains 42 samples where 24 samples are normal and the rest are breast tumor samples. The data was log2 transformed before the application of algorithms. The comparison strategy briefly consists of calculating the ability of different algorithms to differentiate the two sets of samples. This was done by calculating, for each algorithm, the individual recovery scores as given in section 3.2.1, except here term implies the number of conditions common to both biclusters and term implies the number of conditions in their union. Here, the two actual biclusters are: first bicluster contains all genes with first 24 conditions corresponding to normal samples and the second bicluster contains all genes from 25th to 42nd conditions. The output biclusters are biclusters obtained from respective algorithms. The individual recovery scores are plotted for each algorithm in Fig. 4d. Here also, we observed high recovery scores for the CG algorithm in comparison to other algorithms proving its better performance in differentiating the samples.

### Application of the algorithm on a mouse liver microarray data

**Fig. 5:**
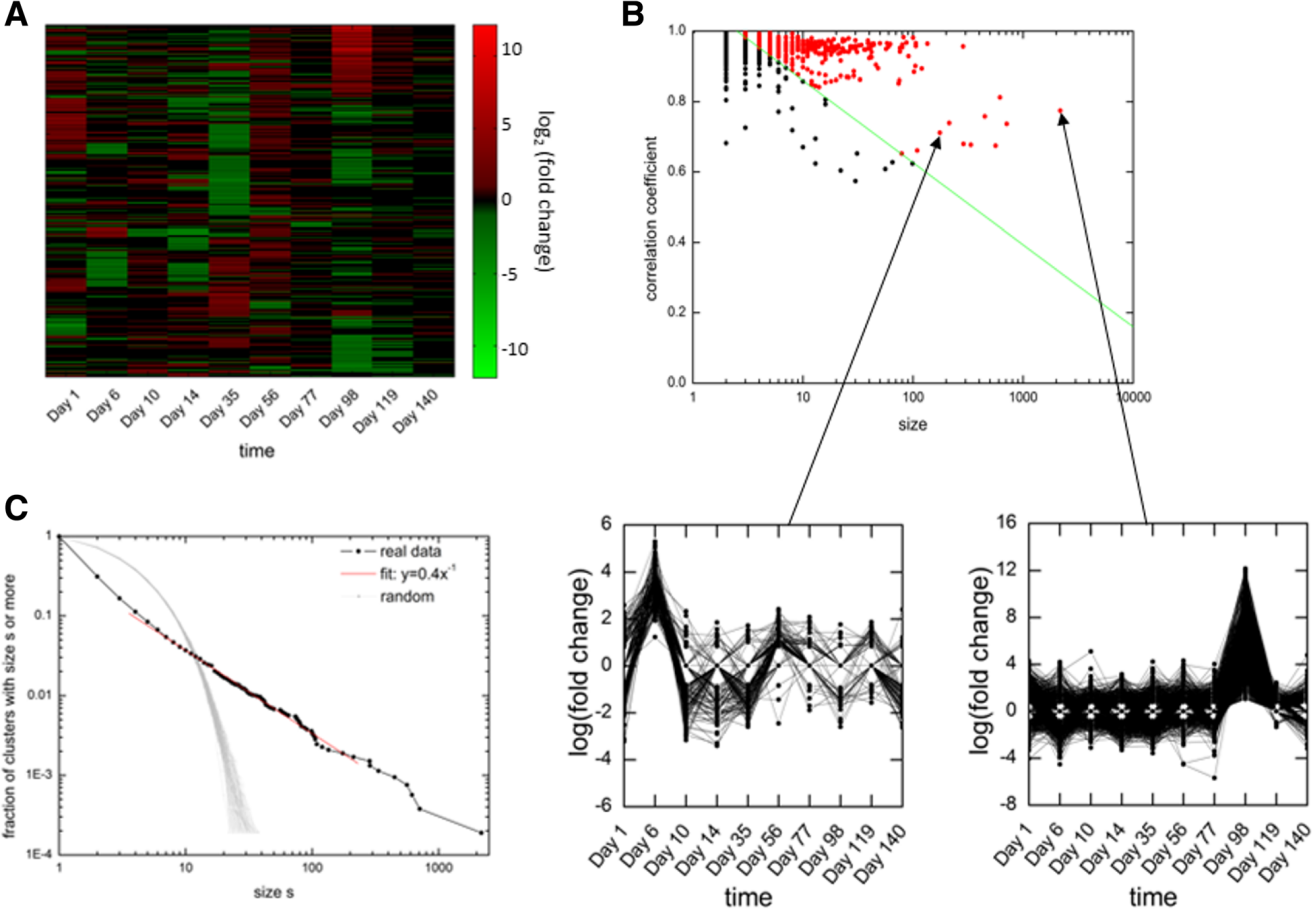
Application of algorithm on a real dataset. **a** A heatmap showing expression levels of genes across time is shown where some cluster of genes up at specific time points can be clearly observed. **b** Correlation coefficient and size of each cluster is shown with two specific clusters’ time profile shown as illustration in below insets. **c** Power law observed in cluster size distribution with slope of −1 with s between 9 to 91 with r2 of .99. Randomly formed clusters behave very differently from the original distribution and thus suggests biologically relevant clustering by the algorithm

After benchmarking our algorithm against predefined biclusters in both synthetic and real datasets, here we will show its application on a real microarray dataset obtained from liver of a mouse fed with high fat high sucrose diet (HFHSD) for different times (hence conditions are time points in this case) (detailed description of data processing and description of data can be obtained in Materials and Methods: Processing of Microarray Data). The microarray data matrix contains log fold change gene expression values for mice under HFHSD fed condition in compared to normal diet condition at different time points. Heatmap of the microarray data matrix is shown in Fig. 5a with genes clustered using clustering algorithm [5]. Some groups of co-expressed genes are clearly visible along with relevant time points, for example, group of genes going up at 8th time point. One can expect metabolic related processes to be perturbed in the data which can be extracted from biclusters as significant processes represented by genes belonging to the biclusters. To filter noisy biclusters that might be present in the data, we used the SELECT function of the algorithm on the resultant biclusters. We calculated the value of c* (used by SELECT function) for *N* = 19303 genes and 10 conditions. The obtained value of c* of 1.093 (Additional file 7: Figure S6A, B), which is very near to the predicted value of c* =1.0887 (obtained as the mean of c* in Fig. 2c). This proves our claim (in section 3.1) that the mean value of c* = 1.0887 can be used for any new dataset without explicitly using random matrix data. Using this c*, we filtered 529 clusters from a total of 5299 clusters obtained from the algorithm (filtered clusters are shown as red dots in Fig. 5b). As an example, we have shown time profiles of genes from two specific clusters (Fig. 5b). The biological processes significantly represented by topmost clusters (Table 1) clearly show that metabolic related processes confirming the result as expected on the basis of the experiment. Finally, to check whether the clusters found by the algorithm are not random, we checked the distribution of the cluster sizes. We observed that the cumulative distribution of cluster sizes follows power law (Fig. 5c), i.e., few clusters with large sizes and large number of clusters with small size. This distribution remains same for the selected clusters too, see Additional file 8: Figure S7. This result is well accordance with other studies, which also observed power law in cluster size distribution [23, 24]. When we plotted distribution for randomly formed clusters (keeping number of clusters (5299) and total cluster size (19303) constant), we observed that it doesn’t follow power law (Fig. 5c). So, the power law distribution confirms that the grouping done by the algorithm is biologically relevant and not random.

**Table 1:** Significant biological processes enriched in some representative clusters. Supporting file is provided separately

Cluster number (size)	Perturbed Time points with sign of up/downreg ulation(+/−)	Biological process	p-value
1 (2179)	8	synaptic transmission (GO:0007268)	3.57E-43
2 (710)	−8	small molecule catabolic process (GO:0044282)	8.97E-06
3 (616)	−5	gene expression (GO:0010467)	6.35E-07
4 (562)	1	embryonic organ development (GO:0048568)	0.0001046
5 (449)	5	transcription from RNA polymerase II promoter (GO:0006366)	1.543E-05
6 (285)	[−8,-5]	translation (GO:00064 12)	1.13E-12

## Discussion

Biclusters play an important role in extracting information from the microarray data, particularly in case if it contains temporal dimension. This can help in elucidating processes perturbed during the experiment under different conditions and can give us mechanistic insights. To extract such biclusters from a microarray data using an algorithm whose input parameters are data independent is a challenging task. In this work, we have developed an algorithm which uses just one user input for generating biclusters. For this, we primarily used the whole time profile of a gene to find the conditions where a gene is expressed. This is similar in concept where the whole time profile of a gene is used to find whether the gene is perturbed or not [14].

In the present study, we have introduced an algorithm to find groups of co-expressed genes and conditions of co-expression. The first advantage of our algorithm is that it is general enough to be used on any kind of high throughput data matrix. It can give output biclusters as overlapping set or as non-overlapping set depending on the choice of the user. Default mode is selecting all biclusters and overlap is allowed. This default mode was used everywhere in our study except in section using Synthetic Dataset 2 and in section with liver and cancer data. In these cases complicated biclusters could come and so it is easier to analyze them as non-overlapping sets. Second advantage is that CG algorithm also doesn’t use any parameter like score cutoff etc., as used by other algorithms. This we could attain by combining the discretization step with the grouping step and hence a single parameter can be used to filter biclusters rather than two parameters usually required for these steps. Finally, applying our novel method of using random matrix data, we have even removed the dependency on this single parameter making our algorithm parameter independent for filtering biclusters.

**Fig. 6:**
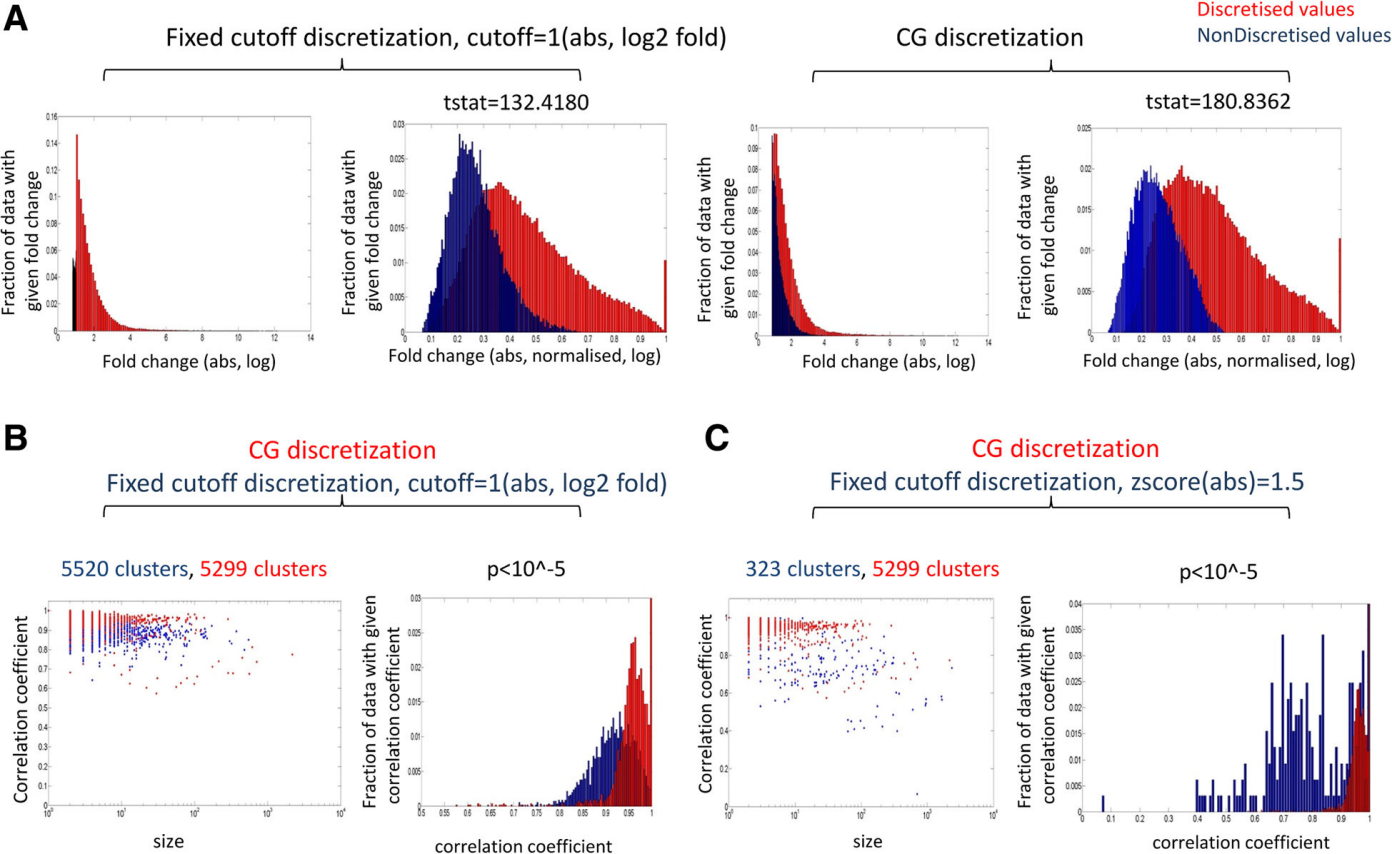
Comparison with other discretization approaches **a** The actual fold change and normalized distribution of discretized and nondiscretized values obtained by applying a 2 fold cutoff and our DISCRETIZE function on the real liver microarray data **b** Correlation coefficient and size of clusters obtained by applying our algorithm on discretized data obtained by using 2 fold cutoff method and our DISCRETIZE function. The distribution of correlation coefficients of clusters is also shown. **c** Correlation coefficient and size of clusters obtained by applying our algorithm on discretized data using our discretization method and zscore cutoff method. The distribution of correlation coefficients of clusters is also shown

Our algorithm discretizes the data without any threshold parameter and gives better results than other algorithms in both synthetic and real datasets as shown by our recovery and relevance analysis. We wanted to explore if we can enhance the performance of our algorithm by applying fixed cutoffs like 2 fold log change or Z-score cutoff [25] in our discretization function. For this, we took a real liver microarray data used in our study and applied a 2 fold cutoff to discretize the data. On plotting the normalized distribution of discretized and non-discretized data, we observed better separation of values in our method as compared to 2 fold cutoff method (statistic using ttest, tstat(CG) > tstat(cutoff)) (Fig. 6a). We further generated clusters using both the methods to see which one is giving more correlated clusters. We found that the clusters obtained using 2 fold cutoff method had significantly less correlation coefficient (p value from ttest < 10^−5^) as compared to clusters generated using the DISCRETIZE function of our algorithm (Fig. 6b). Next, we compared with Z-score cutoff discretization method. For this, we calculated the mean and standard deviation of the expression value of a given gene under different conditions and took the Z-score cutoff of + −1.5 to discretize the data and generated clusters. Here also we found that the clusters obtained using Z-score cutoff method had less correlation coefficient as compared to clusters generated using the DISCRETIZE function of our algorithm (p value from ttest < 10^−5^) (Fig. 6c). Thus, our threshold free discretization method shows better performance than existing fixed cutoff methods.

Here, we derived the score cutoff for the clusters from a matrix by comparing it with randomly generated matrices of same dimension. This means we are deriving score cutoff of clusters assuming all the genes in the original matrix are behaving randomly. This is a very conservative estimate, since in a normal data matrix, all genes won’t be behaving randomly and there would be genes with some definite pattern that would be captured by our algorithm. So, we can safely say that the selected biclusters from the algorithm are not random and are biologically relevant. The algorithm can be applied to any microarray data or other high throughput data like proteomics data to find biclusters.

Since, we have shown that our proposed algorithm performs better in comparison to other algorithms on the dataset with unknown noise levels, so it is expected that the present algorithm will definitely perform better on a dataset with known noise level. Biclusters generated from the algorithm, when integrated with transcriptional networks can help finding transcription factors driving such expression patterns. Also, the selected clusters from two or more microarray datasets can be compared to reveal similarities/differences among the patterns followed by genes of two datasets.

## Conclusions

Biclusters present in a high throughput data is important information to be extracted to find the underlying patterns present in the data. Available biclustering algorithms use many input parameters to find biclusters. Since, on a real dataset, it is difficult to know apriori about the values of these parameters and hence, an algorithm which uses minimum input parameters is highly desirable. We proposed here an algorithm clustered groups, which find groups of co-expressed genes and conditions of co-expression. Despite requiring only a single input parameter, we have shown that our algorithm still works better than other existing algorithms. The algorithm can be used to find such groups in different data types such as microarray (as shown in this study), proteomics, metabolomics etc.

## Additional files

Additional file 1: The matlab code of the algorithm made in the study. Contains the input example small dataset and the output resultant biclusters obtained by application of algorithm on the given small dataset. (XLSX 197 kb)
Additional file 2: Figure S1. Shows the heatmap of the small dataset example and output of each step of our algorithm when applied on this dataset. (PDF 2220 kb)
Additional file 3: Figure S1. Representative examples of gene expression profiles of 8 genes and their discretization. (A-H) The DISCRETIZE step of the algorithm is shown for eight representative genes. Here, the expression level of genes at different conditions taken from an experimental data is plotted in left panels. In right panels, the sorted absolute normalized values of expression data for the respective genes are plotted. Arrows shows the jump above which the expression values are characterized as expressed by the algorithm and the corresponding expression values are shown by circled values here and in actual expression data in left panels in A. This jump is captured by taking the consecutive differences (current minus preceding) of sorted absolute normalized expression profile and identifying the index where this difference is maximum i.e. where jump occurs. (PDF 961 kb)
Additional file 4: Figure S3. Simulations with varying number of genes and conditions. In each of the plot, results for all noise levels and all runs of a fixed dimension input matrix are shown in one plot (A) Each plot depicts the clusters obtained for three noise levels for input matrices of number of genes = 1000 and number of condi-tions equals as given on top of each plot. The value of c* is also shown on the top of each plot. The cluster distribution doesn’t change much with different conditions. (B) Each plot depicts the clusters obtained for three noise levels for input matrices of number of number of conditions = 10 and number of genes equals as given on top of each plot. The value of c* is also shown on the top of each plot. The cluster distribution change and goes towards high cluster size as number of genes in input matrix increases. (PDF 1533 kb)
Additional file 5: Figure S4. Ability of SELECT function to improve relevance of output biclusters (A) Data matrices of size 200 × 100 to 216 × 108 are made corresponding to over-lap degree of 0 to 8 same as in Fig. 3. Here, highest noise level of 0.15 is used. (B) Average recovery scores and average relevance scores for a data matrix of fixed overlap degree corresponding to 10 runs are calculated and mean scores are plotted in figures for QUBIC algorithm and our algorithm with/without SELECT function. No change in recovery scores can be observed and an in-crease in relevance scores are obtained for algorithm with SELELCT function as compared to without SELECT function. (PDF 1431 kb)
Additional file 6: Figure S5. For a matrix with 1000 genes and 10 conditions with expression values of all genes across conditions obtained from normal distribution with mean zero and standard deviation = 0.15, CG algorithm was applied and number of genes of each bicluster(size) is plotted against number of conditions of the corresponding bicluster. As the number of conditions in a bicluster increases, its size decreases. This suggests very less probability of obtaining a large size bi-cluster with large number of conditions. (PDF 298 kb)
Additional file 7: Figure S6. The value of c* as a function of number of genes in the input data matrix. The value of c* for number of genes = 19303 (number of genes present in liver data) predicted using the mean values is 1.0887 which matches with that ob-tained using actual simulation (1.093). (B) Cluster distribution shown using simulation gives value of c* = 1.093. Here, cluster’s correlation coefficient and size obtained for 10 runs of input data matrix of a fixed noise level are overlaid on top of each other. The cluster’s correlation coefficient and size obtained for input data matrices of two other noise levels are also overlaid here. (PDF 704 kb)
Additional file 8: Figure S7. Power law observed in cluster size distribution using only selected clusters shows slope of −1.15 of s between 5 to 74 with r2 of .99. (PDF 506 kb)

## Abbreviations

BICAT: Biclustering Analysis Toolbox
BiMAX: Binary inclusion-maximal biclustering algorithm
CC: Cheng and Church’s algorithm
CG: Clustered Groups
EXPANDER: EXPression ANalyzer and DisplayER
ISA: Iterative Signature Algorithm
QUBIC: Qualitative biclustering
RNA: Ribonucleic acid
SAMBA: Statistical-Algorithmic Method for Bicluster Analysis
TiGER: Tissue-specific Gene Expression and Regulation
