# Supplementary figures and images for: A new method of finding groups of coexpressed genes and conditions of coexpression
Source: BMC Bioinformatics. 2016 Nov 25;17:486. doi: 10.1186/s12859-016-1356-3 (PMC5124285; doi:10.1186/s12859-016-1356-3)

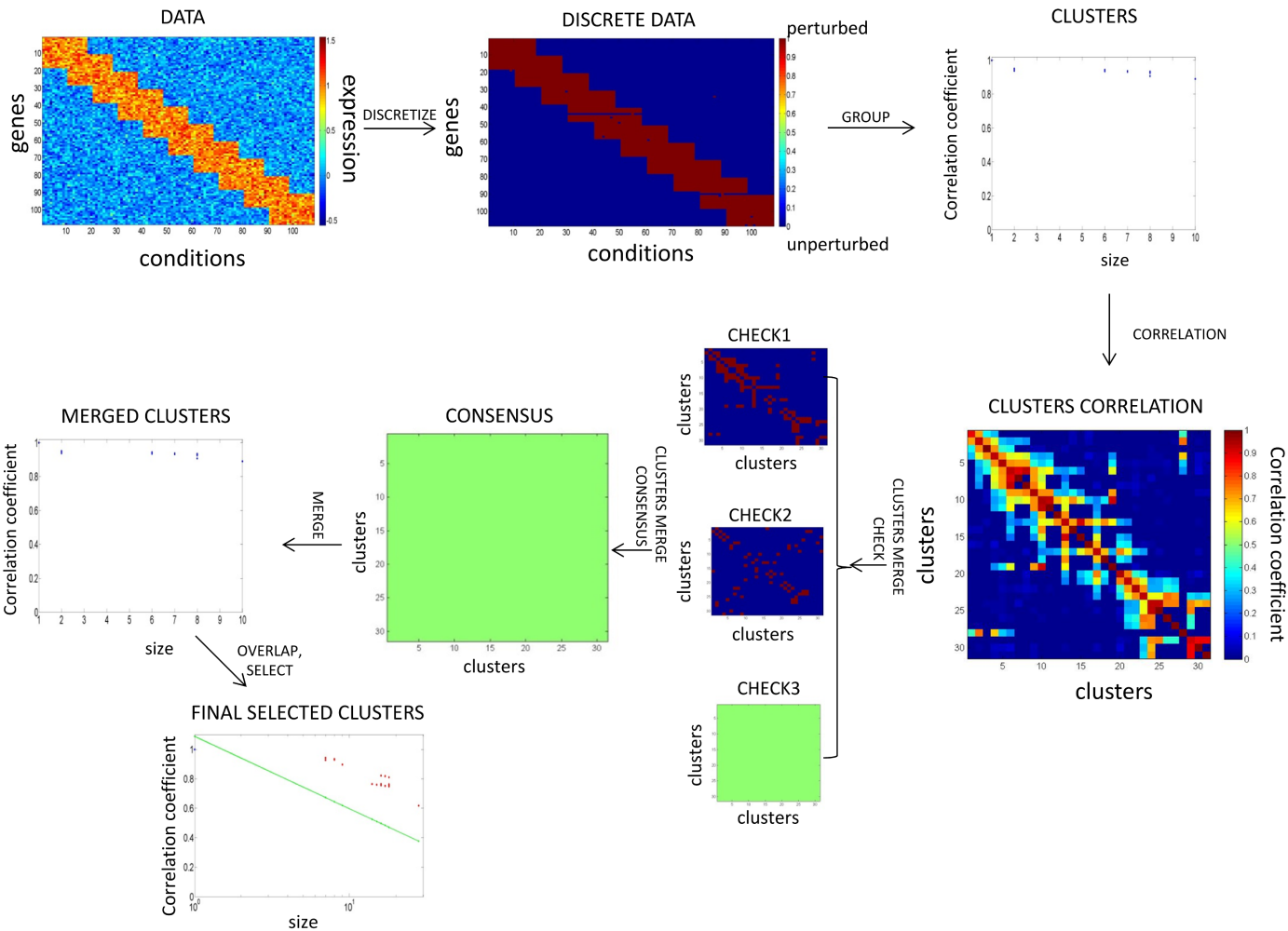

Supplement: Additional file 2: Figure S1. — Shows the heatmap of the small dataset example and output of each step of our algorithm when applied on this dataset. (PDF 2220 kb) [file 12859_2016_1356_MOESM2_ESM.pdf]

A

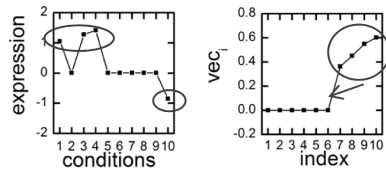

B

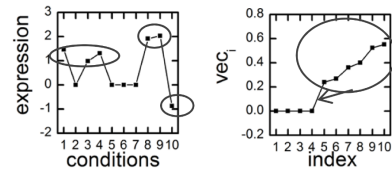

C

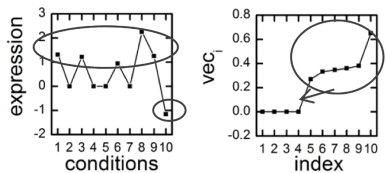

D

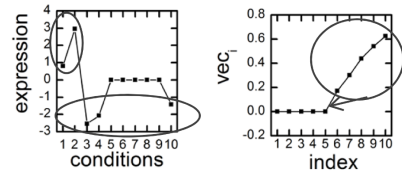

E

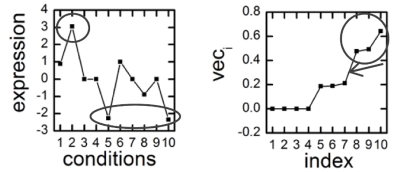

F

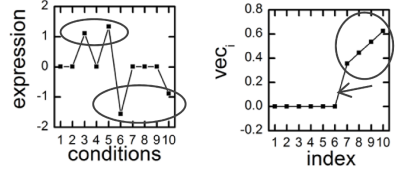

G

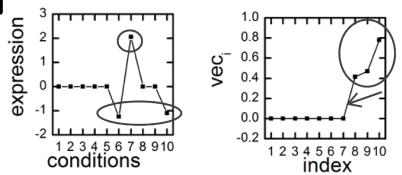

H

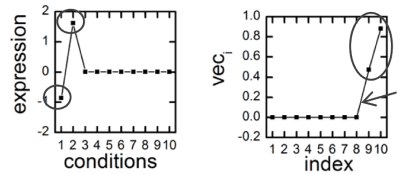

Supplement: Additional file 3: Figure S1. — Representative examples of gene expression profiles of 8 genes and their discretization. (A-H) The DISCRETIZE step of the algorithm is shown for eight representative genes. Here, the expression level of genes at different conditions taken from an experimental data is plotted in left panels. In right panels, the sorted absolute normalized values of expression data for the respective genes are plotted. Arrows shows the jump above which the expression values are characterized as expressed by the algorithm and the corresponding expression values are shown by circled values here and in actual expression data in left panels in A. This jump is captured by taking the consecutive differences (current minus preceding) of sorted absolute normalized expression profile and identifying the index where this difference is maximum i.e. where jump occurs. (PDF 961 kb) [file 12859_2016_1356_MOESM3_ESM.pdf]

A

#genes=1000

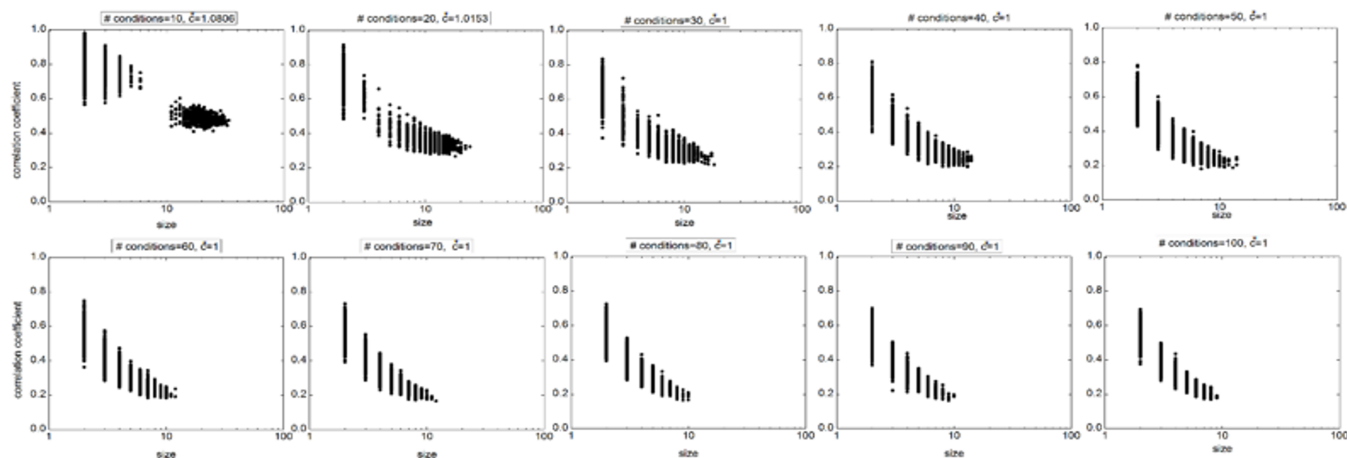

B

#conditions=10

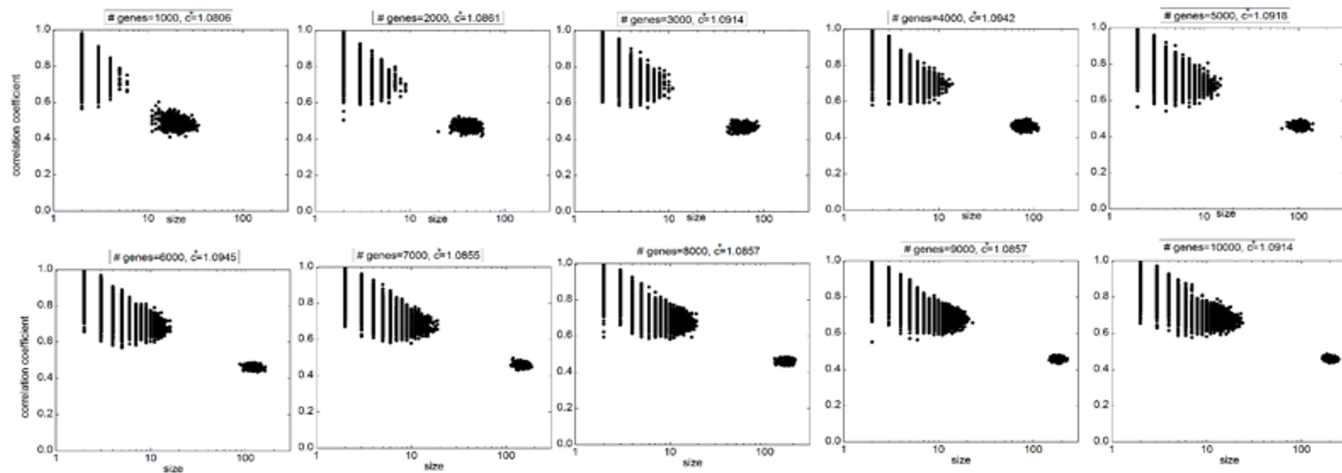

Supplement: Additional file 4: Figure S3. — Simulations with varying number of genes and conditions. In each of the plot, results for all noise levels and all runs of a fixed dimension input matrix are shown in one plot (A) Each plot depicts the clusters obtained for three noise levels for input matrices of number of genes = 1000 and number of condi-tions equals as given on top of each plot. The value of c* is also shown on the top of each plot. The cluster distribution doesn’t change much with different conditions. (B) Each plot depicts the clusters obtained for three noise levels for input matrices of number of number of conditions = 10 and number of genes equals as given on top of each plot. The value of c* is also shown on the top of each plot. The cluster distribution change and goes towards high cluster size as number of genes in input matrix increases. (PDF 1533 kb) [file 12859_2016_1356_MOESM4_ESM.pdf]

A

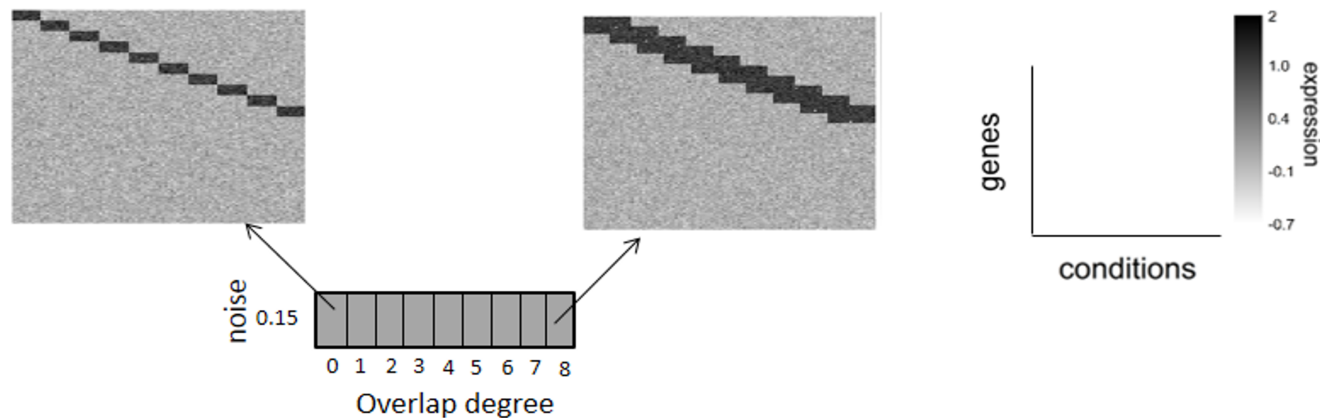

B

Noise=0.15

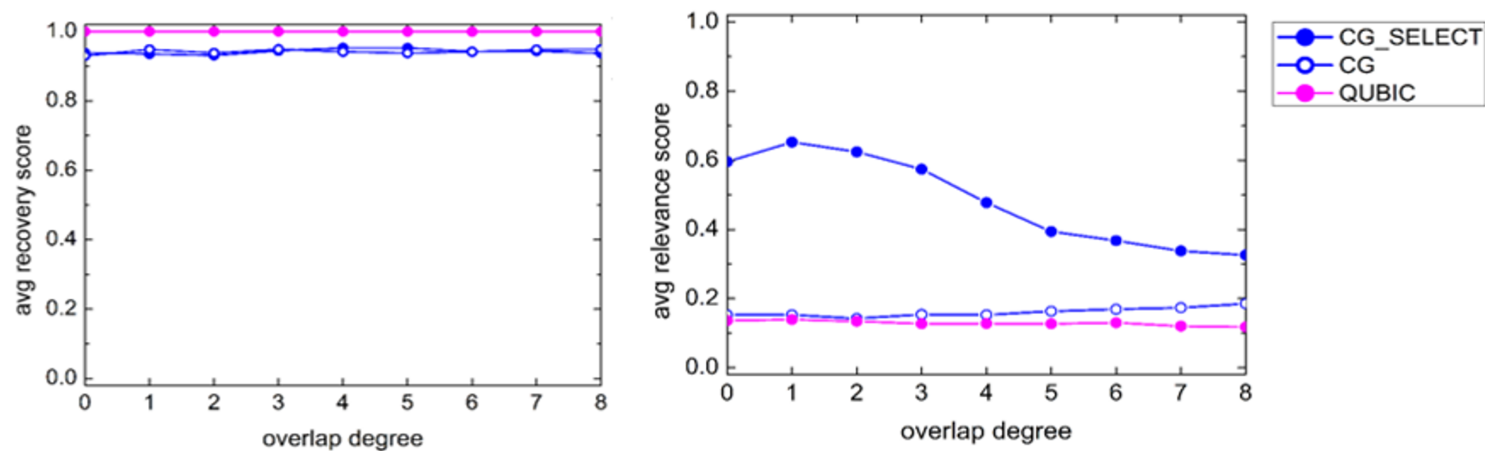

Supplement: Additional file 5: Figure S4. — Ability of SELECT function to improve relevance of output biclusters (A) Data matrices of size 200 × 100 to 216 × 108 are made corresponding to over-lap degree of 0 to 8 same as in Fig. 3. Here, highest noise level of 0.15 is used. (B) Average recovery scores and average relevance scores for a data matrix of fixed overlap degree corresponding to 10 runs are calculated and mean scores are plotted in figures for QUBIC algorithm and our algorithm with/without SELECT function. No change in recovery scores can be observed and an in-crease in relevance scores are obtained for algorithm with SELELCT function as compared to without SELECT function. (PDF 1431 kb) [file 12859_2016_1356_MOESM5_ESM.pdf]

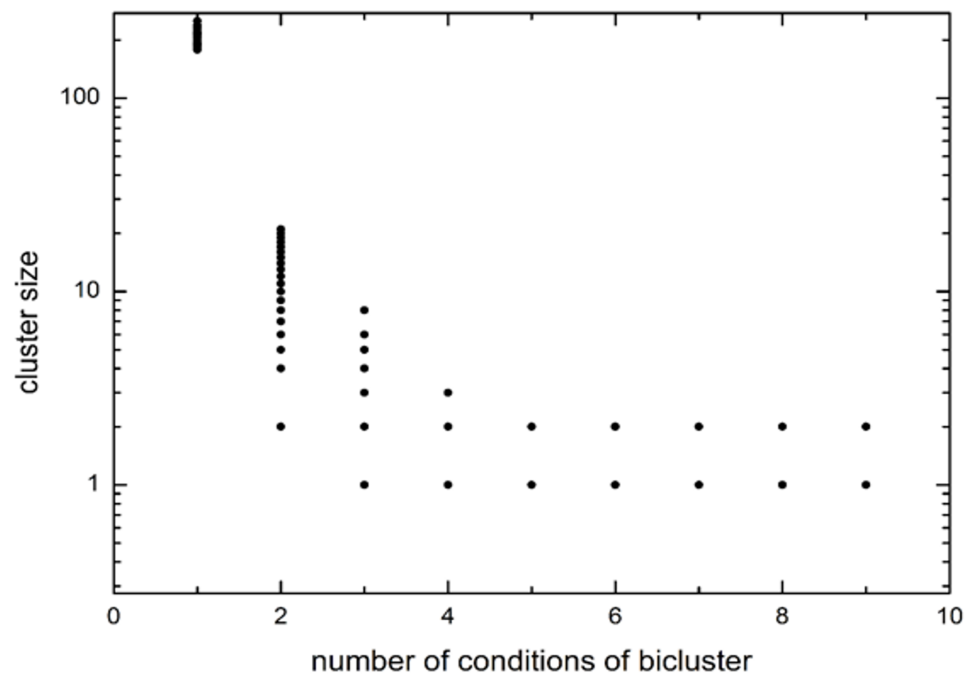

Supplement: Additional file 6: Figure S5. — For a matrix with 1000 genes and 10 conditions with expression values of all genes across conditions obtained from normal distribution with mean zero and standard deviation = 0.15, CG algorithm was applied and number of genes of each bicluster(size) is plotted against number of conditions of the corresponding bicluster. As the number of conditions in a bicluster increases, its size decreases. This suggests very less probability of obtaining a large size bi-cluster with large number of conditions. (PDF 298 kb) [file 12859_2016_1356_MOESM6_ESM.pdf]

**A**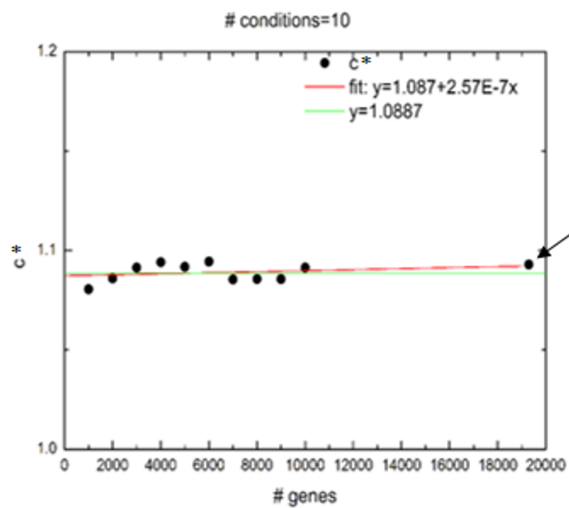**B**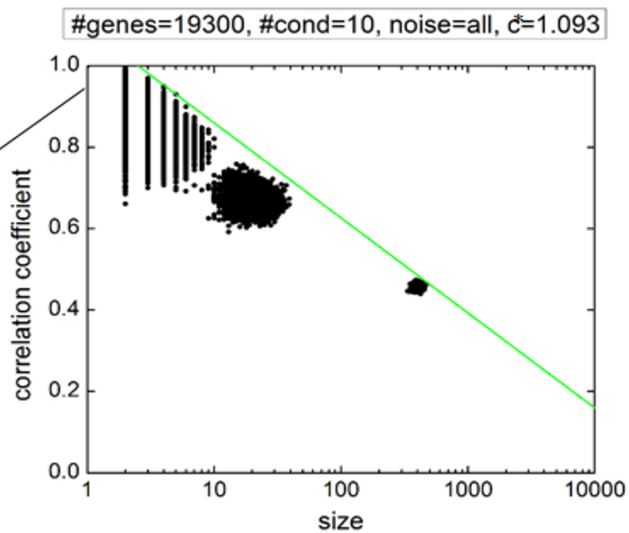

Supplement: Additional file 7: Figure S6. — The value of c* as a function of number of genes in the input data matrix. The value of c* for number of genes = 19303 (number of genes present in liver data) predicted using the mean values is 1.0887 which matches with that ob-tained using actual simulation (1.093). (B) Cluster distribution shown using simulation gives value of c* = 1.093. Here, cluster’s correlation coefficient and size obtained for 10 runs of input data matrix of a fixed noise level are overlaid on top of each other. The cluster’s correlation coefficient and size obtained for input data matrices of two other noise levels are also overlaid here. (PDF 704 kb) [file 12859_2016_1356_MOESM7_ESM.pdf]

A

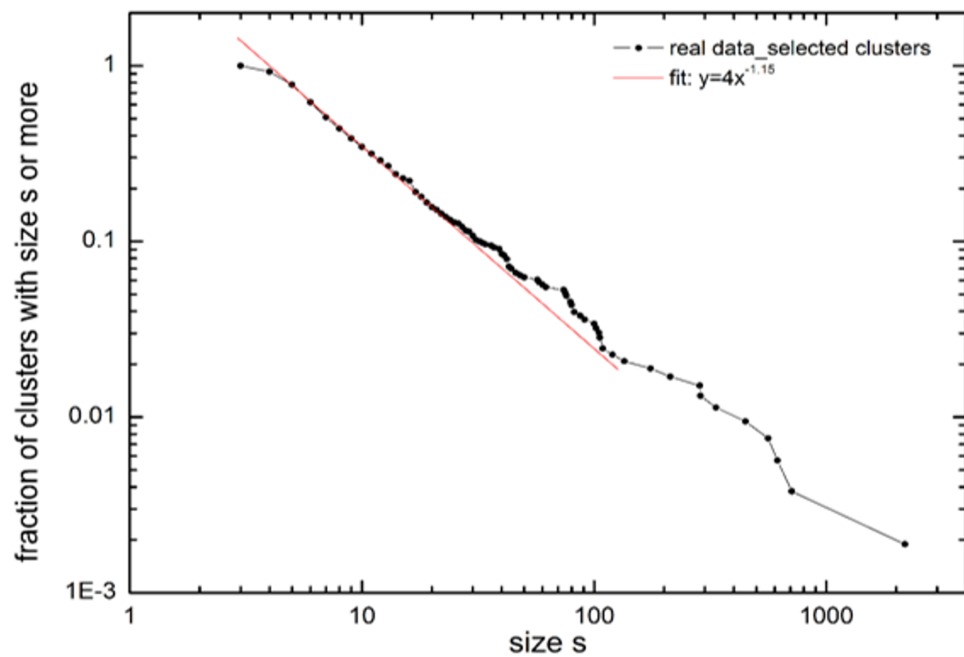

Supplement: Additional file 8: Figure S7. — Power law observed in cluster size distribution using only selected clusters shows slope of −1.15 of s between 5 to 74 with r2 of .99. (PDF 506 kb) [file 12859_2016_1356_MOESM8_ESM.pdf]
